# Disruptions of the intestinal microbiome in necrotizing enterocolitis, short bowel syndrome, and Hirschsprung’s associated enterocolitis

**DOI:** 10.3389/fmicb.2015.01154

**Published:** 2015-10-16

**Authors:** Holger Till, Christoph Castellani, Christine Moissl-Eichinger, Gregor Gorkiewicz, Georg Singer

**Affiliations:** ^1^Department of Paediatric and Adolescent Surgery, Medical University of GrazGraz, Austria; ^2^Department of Internal Medicine, Medical University of GrazGraz, Austria; ^3^Institute for Pathology, Medical University of GrazGraz, Austria

**Keywords:** microbiome, necrotizing enterocolitis, short bowel syndrome, Hirschsprung’s disease, pediatric surgery

## Abstract

Next generation sequencing techniques are currently revealing novel insight into the microbiome of the human gut. This new area of research seems especially relevant for neonatal diseases, because the development of the intestinal microbiome already starts in the perinatal period and preterm infants with a still immature gut associated immune system may be harmed by a dysproportional microbial colonization. For most gastrointestinal diseases requiring pediatric surgery there is very limited information about the role of the intestinal microbiome. This review aims to summarize the current knowledge and outline future perspectives for important pathologies like necrotizing enterocolitis (NEC) of the newborn, short bowel syndrome (SBS), and Hirschsprung’s disease associated enterocolitis (HAEC). Only studies applying next generation sequencing techniques to analyze the diversity of the intestinal microbiome were included. In NEC patients intestinal dysbiosis could already be detected prior to any clinical evidence of the disease resulting in a reduction of the bacterial diversity. In SBS patients the diversity seems to be reduced compared to controls. In children with Hirschsprung’s disease the intestinal microbiome differs between those with and without episodes of enterocolitis. One common finding for all three diseases seems to be an overabundance of Proteobacteria. However, most human studies are based on fecal samples and experimental data question whether fecal samples actually represent the microbiome at the site of the diseased bowel and whether the luminal (transient) microbiome compares to the mucosal (resident) microbiome. In conclusion current studies already allow a preliminary understanding of the potential role of the intestinal microbiome in pediatric surgical diseases. Future investigations could clarify the interface between the intestinal epithelium, its immunological competence and mucosal microbiome. Advances in this field may have an impact on the understanding and non-operative treatment of such diseases in infancy.

## Introduction

The development of the human microbiome seems to start in the prenatal period as fetuses may have contact to microorganisms already *in utero* (Jimenez et al., 2008; Donnet-Hughes et al., 2010; Ardissone et al., 2014). However, the major microbial colonization of the human body starts at birth: During delivery, by passing the mother’s birth canal, the babies acquire a possibly optimized set of microorganisms. Mother’s milk, in addition, provides maternal microbes and prebiotics, which help the gut microbiome to settle and stabilize (Bode, 2009; Hunt et al., 2011). Within the first years of life, the intestinal microbiome of infants undergoes abrupt changes until it reaches a similar status like in adults at approximately three years of age (Palmer et al., 2007; Yatsunenko et al., 2012). The gut microbiome plays an important role during the physiological development: It is extremely important for the differentiation of gut epithelium, interacts closely with the gut-associated lymphoid tissue and influences activity and morphology of the gastrointestinal tract (Cebra, 1999; Cho and Blaser, 2012; Guaraldi and Salvatori, 2012; Hooper et al., 2012; Putignani et al., 2014). Thus, the human development until the age of three is characterized by a highly sensitive interplay between microorganisms and the body. Any disturbances like prematurity, formula feeding, treatment with antibiotics or mode of delivery (Dominguez-Bello et al., 2011; Cho et al., 2012) may have a direct impact on microbial abundance and diversity.

The understanding of the microbiome development seems especially relevant for preterm babies with a gestational age below the 30th week or a very low birth weight (VLBW, <1500 g) (Groer et al., 2014). At this age the physiological interaction between microbes and structural, metabolic, or immunological functions of the gut remains poorly understood. Moreover, there is still a very limited knowledge about the influence of the microbiome on the development of gastrointestinal diseases in early infancy (Groer et al., 2014). Thus, the focus of this review is to provide an in-depth summary of the current knowledge about the gastrointestinal microbiome in infancy with special respect to relevant pediatric surgical diseases such as necrotizing enterocolitis (NEC) of the newborn, short bowel syndrome (SBS), and Hirschsprung’s disease associated enterocolitis (HAEC).

### Necrotizing Enterocolitis

Necrotizing enterocolitis represents a devastating disease primarily affecting premature infants weighing less than 1,500 g. It has been shown that 7% of these infants develop NEC, which carries a mortality rate of up to 30% (Neu and Walker, 2011). Many surviving children subsequently suffer from serious long-term morbidity including intestinal adhesions, bowel resections and even SBS (Pike et al., 2012) making this disease highly relevant for the pediatric surgeon. Despite intense research performed in the field, the exact pathogenesis of NEC has not been revealed yet. Risk factors include gestational age, birth weight, and formula feeding (Stewart and Cummings, 2015). Since NEC does not occur in germ free mice, bacterial colonization seems to play a major role in the development of this disease (Afrazi et al., 2011). In neonates, the intestinal microbiome plays a pivotal role in the development of the epithelial barrier function, integrity and the local and system immune function. Disturbances of the cross-talk between the intestinal microbial community and the immune system may initiate an exaggerated inflammatory response ultimately resulting in NEC (Berrington et al., 2013). The association between bacterial colonization and NEC has been recognized already some decades ago (Neu, 2013). Since then, numerous different bacteria and also viruses have been related to the development of NEC. Until recently, examinations of the intestinal bacterial colonization have been restricted to culture dependent methodologies.

The advent of culture independent technologies, however, has driven research and further deepened our understanding of NEC. There is an increasing number of publications applying molecular sequencing methods comparing intestinal microbial profiles of infants with and without NEC. Many of the available reports have demonstrated disturbances of the intestinal microbiome in infants with NEC. However, the specific findings differ significantly among those studies. Mai et al. (2011) have used high throughput 16S rRNA gene sequencing of stool samples to compare the diversity of microbiota and the prevalence of specific bacterial sequences in nine infants with NEC and in nine matched controls. Interestingly, microbiota composition differed in the matched samples collected one week but not <72 h prior to NEC diagnosis. An increase of Proteobacteria and a decrease in Firmicutes in NEC cases collected one week and <72 h prior to NEC diagnosis were found. Another study with a higher number of patients (11 infants with NEC, 21 matched controls) has demonstrated a tendency toward a lower alpha-proteobacterial diversity in infants, who later developed NEC (Morrow et al., 2013). Furthermore, NEC preceded by Firmicutes dysbiosis occurred earlier (onset days 7–21) than NEC preceded by Proteobacteria dysbiosis (onset days 19–39). The lower bacterial diversity of NEC cases versus controls was confirmed recently (McMurtry et al., 2015). Microbial diversity and *Clostridia* abundance and prevalence even decreased with increasing severity of NEC. One recently published study has investigated genes regulating structural proteins such as tight junctions and cell adhesion in a neonatal rat model of NEC applying a transcriptomic approach (Hogberg et al., 2013). Several tight junction genes such as claudins 1, 8, 14, and 15 and gap junction proteins were found to be involved in the pathogenesis of NEC.

The disruptions of the intestinal microbial profile prior to the development of NEC might open the doors for an early detection and a focused intervention of infants at risk for developing NEC. However, the currently available results are contradictory and inconclusive and support the need of future studies.

Moreover, the microbial composition of fecal samples does not necessarily reflect the situation on the mucosa (Haange et al., 2012). Thus, there is a further need to assess both the physiological development of the microbiome in the different parts of the intestinal tract and potential disruptions in preterm infants developing NEC. A slowly growing body of evidence suggests that in NEC cases there is also a shift of microbes within the intestinal mucosa (Carlisle and Morowitz, 2013). However, no studies have been performed applying deep sequencing techniques on operative specimen of children suffering from NEC compared to control groups. Therefore, further research has to be performed to unravel the influence of the microbiome on the distinct pathogenesis of NEC. Another focus would be to deepen the understanding of microbiome changes prior to NEC development. Such knowledge could foster potential therapeutic strategies including treatment with pre- or probiotics and stool transplantation in order to optimize treatment of infants affected by this devastating disease.

### Short Bowel Syndrome

Short bowel syndrome represents the most common cause for intestinal failure in children. SBS occurs as a congenital disorder or results from surgical removal of diseased gut segments affected by NEC, abdominal wall defects (gastroschisis, omphalocele), midgut volvulus, intestinal atresia, Hirschsprung’s disease, or abnormalities of the superior mesenteric artery (Reddy et al., 2013). The disease-associated loss of absorption capacity of the intestine leads to an inability to maintain fluid, electrolyte, nutrients, or micronutrient balances resulting in a frequent dependency on parenteral nutrition. In a recently published report from a children’s hospital in Canada the incidence of SBS was found to be 22 per 1,000 admissions to the neonatal intensive care unit, which increased further to 43 per 1,000 admissions in premature infants (Wales et al., 2005). The health burden of SBS is significant. A case fatality rate of 27.5–37.5% has been reported within 1.5–5 year follow-up periods in several retrospective studies (Reddy et al., 2013). Failure to thrive (body weight <5th percentile) is seen in more than 70% of patients at 6 months and in almost half of the patients at 2.5 years (Sukhotnik et al., 2002).

The gastrointestinal microbiome of patients suffering from SBS has only been addressed in a very limited number of clinical studies so far (**Table 1**). Using stool and colonic biopsy samples of adult patients with SBS, Joly et al. (2010) have demonstrated that the microbiome of SBS patients is altered (compared to controls) with overabundance of *Lactobacillus* along with a reduced diversity of *Clostridium leptum*, *Clostridium coccoides*, and Bacteroidetes comparing 11 adult patients with SBS to 8 control patients without intestinal pathologies. However, there was no statistically significant microbiome change comparing both groups. SBS-associated microbiota with its high prevalence of *Lactobacillus* is enriched in facultative anaerobic carbohydrate fermenting bacteria (Dethlefsen et al., 2006). Since a considerable amount of fermentable sugars can be consumed by lactobacilli its high prevalence can be interpreted as an expression of the adaptive response of the bowel to SBS (Joly et al., 2009).

**Table 1:** Number of patients, specimen taken, and main findings of human studies investigating the intestinal microbiome in short bowel syndrome (SBS).

	Patients/diagnosis	Specimen	Main findings
Joly et al., 2010	*n* = 11 SBS*n* = 8 controls(adults, monocentric)	Stool samples and biopsies	• Diversity of microbiota associated with colonic mucosa was reduced in SBS compared to controls • Loss of diversity in bacteria from the *Clostridium leptum* group and high numbers of *Lactobacillus* in SBS • Bacteroidetes tenfold lower in SBS
Engstrand Lilja et al., 2015	*n* = 11 SBS (*n* = 5 not weaned from PN)*n* = 7 controls (healthy siblings)(children, monocentric)	Stool samples	• Reduced diversity in SBS children on PN compared to weaned SBS children • High relative abundance of Enterobacteriacae in 6 out of 11 SBS children
Korpela et al., 2015	*n* = 23 IF (out of these *n* = 13 SBS)*n* = 58 controls(multicentric)	Stool samples	• Reduced diversity and richness in IF • Overabundance of lactobacilli, Proteobaceria, and Actionbacteria observed in IF • Proteobacteria were associated with prolonged PN, liver, and intestinal inflammation • Lactobacilli related to advanced liver steatosis mostly after weaning off PN • Liver steatosis, bowel length, PN calories, and antibiotic treatment best explained the microbiota variation of patients with IF

*SBS, short bowel syndrome; PN, parenteral nutrition; IF, intestinal failure*.

Engstrand Lilja et al. (2015) analyzed the microbial profile of 11 children with SBS (5 of them not weaned from parenteral nutrition) and compared the results to seven healthy siblings. One of the major findings was that again diversity (Shannon index) was significantly reduced in children with SBS still on parenteral nutrition compared to weaned children. Additionally, there was a significant overabundance of *Enterobacteriaceae* (belonging to the phylum Proteobacteria) in four out of the five children with SBS on parenteral nutrition. The results demonstrate that intestinal dysbiosis is related to parenteral nutrition in children suffering from SBS. However, it is not clear, whether the observed changes were a cause or a consequence of the disease state. Moreover, four out of the five children with SBS still on parenteral nutrition were treated with antibiotics due to suspected episodes of small bowel bacterial overgrowth (SBBO) at the time of stool sampling. The antibiotic treatment may be a confounding factor since antibiotics have been shown to lower the colonization resistance against *Enterobacteriaceae* by increasing the inflammatory status of the intestinal mucosa (Spees et al., 2013).

Another study also performed an in-depth analysis of the intestinal microbiota in children with intestinal failure using culture-independent phylogenetic microarray analysis (Korpela et al., 2015). An overabundance of lactobacilli, Proteobacteria, and Actinobacteria was observed and the overall diversity and richness were reduced. Proteobacteria, a major phylum of Gram-negative bacteria, were associated with liver steatosis and fibrosis, prolonged parenteral nutrition, and liver and intestinal inflammation in SBS. The lipopolysaccharides (LPS) produced by this Gram-negative strain may explain these results. An experimental approach using TLR-4 knockout mice (LPS insensitive) following small bowel resection could represent an approach to strengthen this assumption.

Further insights into the microbial community changes in SBS may be generated experimentally. Lapthorne et al. (2013) have demonstrated in a piglet model of SBS (75% small bowel resection) a colonic dysbiosis both two and six weeks post-resection. While the total colonic bacterial number (as assessed by absolute quantification using qPCR) showed no significant differences either two weeks or six weeks following small bowel resection, bacterial diversity in the colon was significantly decreased in the resection group at six weeks. Bacteroidetes were decreased and Fusobacteria increased in the resection groups compared to their controls. The majority of differences were observed at family-level within the Firmicutes phylum and a general Firmicutes overabundance leading to an increased Firmicutes/Bacteroidetes ratio at both time points, i.e., 2 and 6 weeks, following small bowel resection. In patients suffering from SBS this ratio and its association with a pro-inflammatory state have not been studied in detail. Moreover little is known about the characterization of the ‘relative microbiota maturity index’ and the ‘microbiota-for-age *Z*-score’, two indices that have been recently described by Subramanian et al. (2014). These two metrics are based on the age-discriminatory bacterial species and a comparison of the observed maturity of a child’s fecal microbiota to healthy children of his/her chronologic age. Therefore, these additional parameters facilitate the classification of undernourished states in children and a possible monitoring tool for applied therapies. The authors have defined a healthy development of the gut microbiome by applying a machine-learning-based approach to 16S rRNA datasets generated from monthly fecal samples. These samples were obtained from a birth-cohort of children living in an urban slum of Dhaka, Bangladesh who exhibited consistently healthy growth. These age-discriminatory bacterial species were incorporated into a model computing the above mentioned indices that compare postnatal development of a child’s fecal microbiota relative to healthy children of similar chronologic age. The model was applied to children with malnutrition of different severities. The obtained results indicated that severe acute malnourishment is associated with significant relative microbiota immaturity that is only partially ameliorated following two widely used nutritional interventions (Khichuri-Halwa and a peanut based ready-to-use therapeutic food). The authors concluded that microbiota maturity indices provide a microbial measure of human postnatal development, a way of classifying malnourished states, and a parameter for judging therapeutic efficacy (Subramanian et al., 2014). Therefore, assessing these indices would be of interest in children suffering from SBS.

Data conflicting the findings of Lapthorne et al. (2013; colonic dysbiosis two and six weeks post-resection) were recently described by Sommovilla et al. (2015) using a murine model. The authors performed a 50% small bowel resection in C57BL6 mice and collected enteric contents from the small bowel, cecum and stool at 7 and 90 days postoperatively for subsequent 16S rRNA gene analysis. No significant changes in bacterial diversity scores of stool, cecal, and ileal samples were found when comparing SBS mice 7 and 90 days following resection to their corresponding sham group. Additionally, no significant community differences at the phylum level at any site of the sampled gastrointestinal tract in the short arm of the study was found. However, at 90 days following small bowel resection the ileal contents significantly differed driven by a decrease in Proteobacteria and Actinobacteria when compared to the respective sham group. Additionally, a comparison of pre- and postoperative (90 days) stool and ileal samples revealed increases of *Lactobacillus* genera. These changes most likely reflect an appropriately adapted community of organisms in response to bowel resection. The beneficial effects of *Lactobacillus* on the gastrointestinal tract include promotion of the innate immunity and its administration has been demonstrated to decrease bacterial translocation (Eizaguirre et al., 2011) and to promote intestinal adaptation (Tolga Muftuoglu et al., 2011). On the other hand, there are cases of complications associated with *Lactobacillus* treatment of children suffering from SBS. Reported complications include bacteremia, sepsis, and D-lactic acidosis (Kunz et al., 2004; De Groote et al., 2005; Munakata et al., 2010). Taken together, there is still insufficient evidence on the effects of probiotics in children with SBS. The safety and efficacy of probiotic supplementation in this high-risk cohort needs to be evaluated in larger trials. The authors of the abovementioned study explained the absence of differences in the overall bacterial diversity throughout the gastrointestinal tract by the fact that unlike other studies perioperative antibiotics were not used and antibiotics lead to a reduced microbial diversity (Dethlefsen and Relman, 2011). Additionally, a 50% small bowel resection might retain sufficient intestinal length in this murine model. In contrast to the findings of Sommovilla et al. (2015), a decreased bacterial diversity in the colon in a murine model of ileocecal resection (removal of 12 cm ileum, cecum, and proximal right colon) and accompanying antibiotic treatment has been described recently (Devine et al., 2013).

In patients suffering from SBS, parenteral nutrition, the frequent use of antibiotics due to recurrent infections and the accelerated intestinal transit time may all lead to substantial clinical problems (Kaneko et al., 1997). The altered microbiome-gut homeostasis in SBS leads to a disrupted gastrointestinal barrier function and capacity of the microbiota to provide vitamins and their precursors contributing to the frequent complications seen in SBS patients such as sepsis, vitamin deficiencies and failure to wean from parenteral nutrition (Sommovilla et al., 2015). Moreover, SBBO, defined as an increase of the total number of bacteria per ml content, may develop in children with SBS. SBBO is caused by stasis, poor peristalsis, and intestinal dilatation. Clinically SBBO significantly increases the risk for recurrent blood stream infections and the systemic proinflammatory response decreases with increasing enteral feeding and weaning parenteral nutrition (Cole et al., 2010).

Taken together, microbiome research of SBS is still in its infancy. A marked dysbiosis of the gastrointestinal tract seems to be characteristic for patients suffering from SBS. The presently available data based on next-generation sequencing indicate that the disruptions of the gastrointestinal microbiome may be a reason for the pro-inflammatory state associated with SBS. Nevertheless, the precise alterations of the microbiome associated with SBS, i.e., changes of the transient/resident microbiome during intestinal adaptation processes, the physiological and pathophysiological roles of the altered microbes and safe possibilities of therapeutic manipulations have still to be unraveled.

### Hirschsprung’s Associated Enterocolitis

Hirschsprung’s disease describes a congenital segmental absence of the enteral nervous system (ENS) in the myenteric and submucosal plexuses with variable proximal expression due to a failure of migration of neural crest cells during embryonic development (Sasselli et al., 2012). The resulting intestinal obstruction is usually treated by surgical removal of the aganglionic bowel and a pull-through of unaffected ganglionic bowel.

Despite correct removal of the aganglionic bowel, up to 40% of the patients continue to suffer from Hirschsprung’s associated enterocolitis (HAEC; Frykman and Short, 2012). HAEC is defined as a clinical condition with explosive diarrhea, abdominal distension, fever, and subsequent septic shock (Elhalaby et al., 1995). Immediate treatment of HAEC patients with bowel rest, rectal washouts, adequate resuscitation, and antibiotic treatment has decreased its mortality to about 1%. Even though a variety of different hypotheses have been formulated, the exact pathophysiology of HAEC is still unclear. Recent studies have shown that a disruption of the intestinal mucosal barrier, an abnormal immune response of the intestinal tract and infection due to specific pathogens like *Clostridium difficile* may play pivotal roles in the development of HAEC (Hong and Poroyko, 2014). Considering the interrelation between the epithelium, the immune system and the microbiome of the intestine, disturbances of the intestinal microbial composition may predispose a patient to develop HAEC independent of correct surgical treatment.

Both, experimental and clinical studies recently have given a first insight into an altered intestinal microbiome in Hirschsprung’s disease and HAEC. Applying 16S rRNA gene pyrosequencing Ward et al. (2012) assessed the intestinal microbiome in a murine model of HAEC for the first time. The authors used endothelin receptor B knockout mice (Ednrb –/–) as an established experimental model of intestinal aganglionosis. Colonic and fecal samples were analyzed at different time points and compared to wild type mice (WT). WT exhibited increasing species diversity with age, while mutant mice possessed an even greater diversity. On the phylum level, mutant mice contained more Bacteroidetes and less Firmicutes compared to WT mice. Based on these results the ENS can be added to the list of regulatory host factors influencing microbial composition. These findings were – at least partially – confirmed by a study comparing bacterial DNA of cecal contents between Ednrb knockout and heterozygous mice (Pierre et al., 2014). The heterozygous mice demonstrated decreased levels of Bacteroidetes and Proteobacteria with increased Firmicutes compared to the knockout mice. Additionally, mutant mice exhibited a reduced ileal expression and activity of secretory phospholipase A_2_ (sPLA_2_), an antimicrobial molecule secreted by Paneth cells at the base of the intestinal crypts. These results suggest that Hirschsprung’s disease is not limited to the absence of ganglia within the gastrointestinal tract, but also affects the mucosal immune system and subsequently the microbial composition of the GI tract.

De Filippo et al. (2010) assessed 15 stool samples of a 3-years old Hirschsprung patient harvested during four HAEC episodes and remission phases, respectively. The samples were analyzed using amplified ribosomal DNA restriction analysis (ARDRA). This analysis revealed that HAEC episodes clustered together suggesting a sort of predisposing bacterial community for HAEC development. The available human studies on HAEC applying high-throughput sequencing are summarized in **Table 2**. Yan et al. (2014) assessed the microbial signature of intestinal contents taken during surgery from different sections along the intestinal tract in a study population consisting of four patients (two patients with HEAC and two patients with Hirschsprung’s disease). Bacteroidetes and Proteobacteria accounted for the highest proportion among the intestinal flora in Hirschsprung’s patients. In contrary, Proteobacteria and Firmicutes were the most common microbes in HAEC patients. At the genus level, marked differences comparing HAEC and Hirschsprung’s patients were observed. The altered Firmicutes/Bacteroidetes ratio found in this study confirms the above-mentioned changes described in experimental settings and fuels the speculation that the increased ratio is associated with a pro-inflammatory state of the intestinal microbiomes. The alterations of the dominating bacterial phylum reduce the endogenous production of GLP-2, an intestinal peptide enhancing tight junctions of the cells and thereby preventing LPS from entering plasma (Cani et al., 2009). In a larger group of patients suffering from Hirschsprung’s disease consisting of 19 children, 9 with a history of HAEC and 9 without, fecal DNA was isolated and the bacterial and fungal microbiome was analyzed subsequently (Frykman et al., 2015). Even though bacterial microbiome analysis revealed some differences between the two groups at the phylum level, the changes did not reach statistical significance. In contrast, the fecal fungal composition (the mycobiome) of the HAEC group showed a marked reduction in diversity with increased *Candida* sp. and reduced *Malassezia* and *Saccharomyces* sp. compared with the group of patients without HAEC. These results therefore identified *Candida* sp. as a potential player in HAEC, either as an expanded commensal species as a consequence of enterocolitis or its treatment or even possibly contributing to the pathogenesis of HAEC (Frykman et al., 2015). Once more it should be emphasized that fecal samples may not reveal the microbial diversity of the diseased bowel and even luminal samples may not represent the mucosal microbiome (Haange et al., 2012). Thus, further research seems to be mandatory.

**Table 2:** Number of patients, specimen taken, and main findings of human studies investigating the intestinal microbiome in Hirschsprung’s disease (HD) and Hirschsprung’s associated enterocolitis.

	Patients/diagnosis	Specimen	Main findings
Yan et al., 2014	*n* = 2 HAEC*n* = 2 HD(children, monocentric)	Intestinal contents from different sections along the intestine during operative procedure	• HAEC patients exhibited greater diversity of bacterial species than HD patients • Sharp distinction between microbiota of HAEC and HD • Phylum level: Proteobacteria (55%) and Firmicutes (18%) in HAEC; Bacteroidetes (46%) and Proteobacteria (21%) in HD • Genus level: *Enterobacteriaceae* (56%) in HAEC; *Bacteroides* (47%) in HD
Frykman et al., 2015	*n* = 9 HAEC*n* = 2 HD(children, multicentric)	Stool samples collected after completed definitive surgery	• HAEC patients exhibited greater diversity of bacterial species than HD patients • No significant differences in the proportion of bacterial groups at the phylum and genus level • Reduced diversity of fungal species in HAEC compared to HD • Increased *Candida* sp. and reduced *Malassezia* and *Saccharomyces* sp. in HAEC • High burden of *C. albicans* in some HAEC patients, not found in HD patients

*HAEC, Hirschsprung’s associated enterocolitis; HD, Hirschsprung’s disease*.

Taken together, there is a limited but growing body of evidence that a shift in the intestinal microbiome with respect to the colonization with specific intestinal bacteria may affect the intestinal immune responses causing a susceptibility to recurrent life-threatening episodes of HAEC. Additionally, it has already been shown that an altered transit time due to disruptions of the intestinal motility is an important factor for shaping the intestinal microbiome (Gorkiewicz et al., 2013). Nevertheless, it still seems too early to recommend possibilities to alter the intestinal microbiome in a therapeutic way. Further studies have to be performed to reveal the crosstalk between the intestinal microbiota and the immune system.

## Discussion and Future Perspectives

Especially in neonates or preterm babies the immunological, structural, and metabolic interaction between the microbiome and the gastrointestinal tract is not completely understood yet. Latest (and future) 16S rRNA gene-based and metagenomic analyses may provide novel insights into the development of the gastrointestinal microbiome in such patients. In addition to genome-based information, functional data (assessed via transcriptomics or metabolomics) of the infant microbiome need to be retrieved allowing the vision of an improved diagnosis and treatment of infectious diseases in the future.

At present we already have scientific evidence for diseases like NEC, SBS, and HAEC that dysbiosis of the microbiome may influence the course of the diseases. Further insights into the molecular interaction between the microbes and the gut, as well as high-resolution visualization of the interplay seem mandatory as present data are not conclusive.

One challenge associated with studies of the microbiome in pediatric surgical diseases like NEC, SBS, and HAEC is the low incidence of these diseases. The resulting low numbers of individuals combined with the presence of potential confounding factors make interpretation of “microbiome data” challenging. Confounding factors include mode of delivery and feeding (breast milk vs. formula; enteral vs. parenteral). For instance, in the case of caesarian delivery a different set of environmental bacteria (similar to the skin communities of the mothers) form the basis for the infant’s microbiome compared to the microbiome of infants delivered vaginally (Biasucci et al., 2008; Dominguez-Bello et al., 2010). Moreover, while the microbiome of breast-fed infants is dominated by bifidobacteria the counts of *Escherichia coli*, *Clostridium difficile*, *Bacteroides fragilis*, and lactobacilli are higher in exclusively formula-fed infants (Penders et al., 2006). In a recently published study it has also been shown that immaturity and perinatal antibiotics strongly affected the infant’s microbiome (Arboleya et al., 2015).

Nutrition as well as environmental conditions of the neonatal intensive care unit can also strongly influence the microbial development. For example, it has been shown that human milk carries pre- and probiotics, which are necessary for an ideal colonization of the gut and could thus, help pre-term and low-weight infants to develop optimally (Sela and Mills, 2010).

Moreover, the environment seems to matter. Recent studies indicate that (neonatal) intensive care units harbor hot spots of possibly pathogenic microorganisms located on the medical personnel and equipment (Gastmeier et al., 2007; Oberauner et al., 2013). Due to the frequent and harsh cleaning processes in such areas, the natural microbial community is strongly influenced and shifted toward a potentially more resistant microbiome, as it has also been observed for highly cleaned and monitored clean rooms (Moissl-Eichinger et al., 2013). An overview summary of neonatal intensive care unit outbreaks has identified *Klebsiella* sp. to be responsible for most outbreaks. This bacterium, which is widely distributed in various habitats including the sinks of patient rooms (Leitner et al., 2015), possesses a polysaccharide capsule, which makes this microbe more robust against desiccation – a critical feature to survive on surfaces or on skin.

Finally the babies age matters. Brooks et al. (2014) have shown that VLBW infants adopt microorganisms from their hospital environment. Microbial reservoirs in the rooms inoculate the infant’s intestinal tract and thus impact the development of the microbiome. From there, the microorganisms are distributed again into the environment, creating a cycle of inoculation. In this study, the most probable reservoirs for different microorganisms were found to be tubings and surfaces, whereas hands or skin contributed to a lesser extent.

## Conclusion

The interplay of the microorganisms with VLBW infants is extremely complex, and understanding the processes and finding possibilities to control this sensitive interaction requires are large (international) collaboration. With the joint goal to understand the impact of the microbiome on infant development also geographical differences between regions for the same disease can be studied, with the chance to improve health and development for generations of VLBW- and hospitalized newborns. However, collecting fecal samples may not be sufficient to define the microbiome of the diseased bowel, because Haange et al. (2012) have shown in an experimental setting that the microbial diversity differed considerably along the intestinal tract and even the luminal (transient) microbiome displayed a different diversity compared to the mucosal (resident) microbiome at the same portion of the gut. Finally, epidemiological studies beyond infancy seem promising to observe the “normal development” of the microbial diversity (e.g., in the appendix) and ensure a protective and beneficial symbiosis.

## Conflict of Interest Statement

The authors declare that the research was conducted in the absence of any commercial or financial relationships that could be construed as a potential conflict of interest.
